# Supplementary material for: EnzML: multi-label prediction of enzyme classes using InterPro signatures
Source: BMC Bioinformatics. 2012 Apr 25;13:61. doi: 10.1186/1471-2105-13-61 (PMC3483700; doi:10.1186/1471-2105-13-61)
Supplement: Addtional file 5 — The Java code to format the data files, evaluate and predict. The file enzml_java_code.tar.gz contains the Java code used to format database data to ARFF and XML formats, to execute cross and train-test (jackknife) evaluations and to record evaluation results to database. More information is included in the readme.txt file and the Javadoc files. The code can be used with a MySQL database. To use a different database software, other JDBC drivers might be required. [file 1471-2105-13-61-S5.gz › java_code/utils/doc/uk/ac/ed/inf/utils/maputils/IndexedOneToManyMap.html]

IndexedOneToManyMap


---


|  |  |  |
| --- | --- | --- |
| **Overview** \| **Package** \| **Class** \| **Use** \| **Tree** \| **Deprecated** \| **Index** \| **Help** | |  |
| PREV CLASS   **NEXT CLASS** | **FRAMES**    **NO FRAMES**     **All Classes** |  |
| SUMMARY: NESTED \| FIELD \| CONSTR \| METHOD | DETAIL: FIELD \| CONSTR \| METHOD |  |


---


## uk.ac.ed.inf.utils.maputils Class IndexedOneToManyMap<T,U>

```
java.lang.Object
  uk.ac.ed.inf.utils.maputils.OneToManyMap<T,U>
      uk.ac.ed.inf.utils.maputils.IndexedOneToManyMap<T,U>
```

---

``` public class IndexedOneToManyMap<T,U> extends OneToManyMap<T,U> ```

A one-to-many map that uses an internal integer index to save memory. Useful,
for example, for maps having few, long-string repeated values. Do not use if
you need to add or remove individual objects.
This map cannot take an empty or null string as key or value.

**Version:**
:   28 Mar 2011

**Author:**
:   Luna De Ferrari luna.deferrari-at-ed.ac.uk

---

**Field Summary**

**Fields inherited from class uk.ac.ed.inf.utils.maputils.OneToManyMap**

`m_map`


**Constructor Summary**

`IndexedOneToManyMap()`
  
          Initialise an empty map

`IndexedOneToManyMap(java.util.Vector<T> keys,
java.util.Vector<U> values)`
  
          Initialise with a list of key-value pairs


| **Method Summary** | |
| --- | --- |
| `boolean` | `containsKeyValue(T key, U value)`             Checks if the map contains a specific key-value pair |
| `boolean` | `containsValue(U value)`             Checks if the map contains a value |
| `java.util.Set<U>` | `getAKeyValues(T key)`             Get the set of unique values for this key. |
| `java.util.Set<U>` | `getNonEmptyValues()`             Get the set of unique, non-empty-string values for this map. |
| `java.util.Set<U>` | `getValues()`             Get the set of unique values for this map. |
| `void` | `initialise()`             public TreeMap> getIndexedMap() { return m\_map; } |

**Methods inherited from class uk.ac.ed.inf.utils.maputils.OneToManyMap**

`addAll, addKeyAndValue, containsKey, deleteKeyValue, getKeys, getKeysVectorValuesVector, getKeyValuesPairsSize, getMap, getNonEmptyKeys, getNonEmptyKeyValuesPairsSize, mapIsNullOrEmpty, size, toCSV, toString`

**Methods inherited from class java.lang.Object**

`equals, getClass, hashCode, notify, notifyAll, wait, wait, wait`

**Constructor Detail**

### IndexedOneToManyMap

```
public IndexedOneToManyMap()
```

:   Initialise an empty map

---


### IndexedOneToManyMap

```
public IndexedOneToManyMap(java.util.Vector<T> keys,
                           java.util.Vector<U> values)
```

:   Initialise with a list of key-value pairs

    **Parameters:**: `keys` - the list of keys: `values` - the list of values (the value for each key in the keys list)


**Method Detail**

### containsKeyValue

```
public boolean containsKeyValue(T key,
                                U value)
```

:   Checks if the map contains a specific key-value pair

    :   **Overrides:**: `containsKeyValue` in class `OneToManyMap<T,U>`
    :   **Parameters:**: `key` -: `value` - **Returns:**: true if the key-value exists, false otherwise (false if the key or value are null)

---


### containsValue

```
public boolean containsValue(U value)
```

:   Checks if the map contains a value

    :   **Overrides:**: `containsValue` in class `OneToManyMap<T,U>`
    :   **Parameters:**: `value` - a value **Returns:**: true if the value exists in the map, false otherwise

---


### getAKeyValues

```
public java.util.Set<U> getAKeyValues(T key)
```

:   Get the set of unique values for this key. No values can be null. At most
    one of the values can be an empty string.

    :   **Overrides:**: `getAKeyValues` in class `OneToManyMap<T,U>`
    :   **Returns:**: the set of unique values for this key

---


### getValues

```
public java.util.Set<U> getValues()
```

:   Get the set of unique values for this map. At most one value can be null.
    At most one value can be an empty string.

    :   **Overrides:**: `getValues` in class `OneToManyMap<T,U>`
    :   **Returns:**: the set of unique values for this map

---


### initialise

```
public void initialise()
```

:   public TreeMap> getIndexedMap() { return m\_map; }

    :   **Overrides:**: `initialise` in class `OneToManyMap<T,U>`

---


### getNonEmptyValues

```
public java.util.Set<U> getNonEmptyValues()
```

:   Get the set of unique, non-empty-string values for this map.

    :   **Overrides:**: `getNonEmptyValues` in class `OneToManyMap<T,U>`
    :   **Returns:**: the set of unique, non-empty-string values for this map


---


|  |  |  |
| --- | --- | --- |
| **Overview** \| **Package** \| **Class** \| **Use** \| **Tree** \| **Deprecated** \| **Index** \| **Help** | |  |
| PREV CLASS   **NEXT CLASS** | **FRAMES**    **NO FRAMES**     **All Classes** |  |
| SUMMARY: NESTED \| FIELD \| CONSTR \| METHOD | DETAIL: FIELD \| CONSTR \| METHOD |  |


---
